# Cases

**Published:** 1829-04

**Authors:** Edward Barcome

**Affiliations:** George Town, Demerara.


THE LONDON
### Medical and Physical Journal.

Nº 362, VOL. LXI.] APRIL, 1829. [Nº 34, *New Series.*

For many fortunate discoveries in medicine, and for the detection of numerous errors, the world is indebted to the rapid circulation of Monthly Journals; and there never existed any work, to which the Faculty, in Europe and America, were under deeper obligations than to the *Medical and Physical Journal of London,* now forming a long, but an invaluable series.—RUSH.

### ORIGINAL PAPERS, AND CASES,

### OBTAINED FROM PUBLIC INSTITUTIONS AND OTHER AUTHENTIC SOURCES.

### DISEASES IN DEMERARA.

*Cases;* by EDWARD BARCOME, Esq. George Town, Demerara. (Communicated by Dr. JAMES JOHNSON.)

*Colica Pictonum.*

WILLIAM LYNCH, aged twenty-seven, of a spare habit, by trade a tailor, states that on Friday last (five days back), while at breakfast, he felt a disinclination to eat, and nausea; soon after, his bowels became affected with slight wandering pains, and, on going to the privy, he could not evacuate any thing. He took a dose of salts; which was repeated, without producing any effect on the bowels. The symptoms increased, and some rhubarb and calomel was taken: still no evacuation from the bowels. Vomited, soon after swallowing the powder, a large quantity of a greenish and bitter fluid.

Is now suffering greatly, tossing about in the bed, and cannot remain a moment in one position; says that he feels as if his belly will burst; cannot bear the slightest pressure on the abdomen, which is very tense. Headach; thirst insatiable; tongue white, and coated with mucus; mouth parched and dry; acid eructations; difficult micturition; urine high coloured; pulse full, tense, and frequent; temperature below the natural standard; cold clammy sweat; nausea, and vomiting of a greenish and bitter fluid. Has not slept for six nights; great prostration.

Fiat venæsectio ē brachio, stat. ut sang. emittatur ad ℥x. dein applicetur scarificator, epigast. et region. umbilic. ad extract. sang. ℥xviij. Foment. calid. toto abdomini. Adhibeantur enem. emollient. omni semi-hora donec alvus bene respond. et habeat pro potu ordinario ℔ decoct. hordei, &c.

Two P.M.—Says that he feels something easier; thirst not so urgent. The sixth enema, on coming away, was discoloured in a trifling degree. Abdomen less tense, but painful on pressure; pulse more natural; skin cool and comfortable.

Iterum appl. scarificator, toto abdomini ut sanguis extrahatur ad ℥xxx.; et contiuantur foment. potus enemataque.

Eight P.M.—Bowels have been evacuated six times since last visit: a large quantity of very fetid matter discharged, having the appearance of oil and indurated feces. Says that he does not feel any pain, but a soreness, as if his belly had been scraped inside. Pulse soft, but rather frequent; thirst moderate; urine high coloured, but passed without difficulty. Perspires freely.

Cont. potus foment. enemataque.—℞. Extract. Opii gr. ij.; Pulv. Jacobi gr. iss. M. fiat pilula, hora somni sumenda.

October 24th, morning.—Bowels have been evacuated four times since evening, evacuations more natural. Pulse regular; temperature natural; thirst moderate; tongue clean and moist. Says that he feels comfortable; has slept about four hours the last night.

Cont. potus enemataque sed omitt. foment.

25th.—Is convalescent.

26th.—Improving. Ordered to continue on low diet for a week: arrow root, jelly, sago, light soups, &c.; and to open the bowels, if necessary, with emollient enemas.

*Reflections on the foregoing Case*; by Staff-Surgeon DOYLE, Bermuda.

It is to be lamented that nothing is said by the practitioner of the idiosyncrasy of the patient: whether he was of the sanguine, bilious, or nervous temperament; whether irritable, calm, or phlegmatic; what complexion, &c.; what previous malady or affection he had undergone, &c.: for all these are of importance to etiology, and to the study of the excitation of the different organs, if, as Bordeu and Bichât have told us, all the tissues of which the animal economy is composed have each of them a peculiar mode of action; a life, in fact, peculiar to itself. This action is susceptible of aberration; and it is in this that all pathology must consist, says Broussais. Now, in order to render this important case instructive and profitable to medical science, it will be best to subject the group of symptoms which characterizes it to a rigid analysis, in order thereby to arrive at a knowledge of its true nature.

It appears that five days before (23d October,) this patient had a predominance of (morbid) irritation in the *stomach*, a viscus capable of exciting the greatest number of sympathies; for sensibility and contractility being distributed in different degrees in the divers tissues which make up the living organism, those which possess it in the highest degree receive the immediate action of stimulants, and transmit it to others. They are, for this reason, the natural movers of sympathies. The gastric disturbance was manifested by anorexia and nausea: this disturbance is rapidly communicated to the intestines, both smaller and larger; for, the more the sensibility of the irritated organ and that of the individual are considerable, the more the sympathies are multiplied, and vice versa. This propagation of the gastric irritation into the intestines occasions the wandering pains in the bowels and the desire to go to stool. The morbid congestion having invaded the ileo-cœcal valve, the ileum cannot force its stercoral contents through it: hence costiveness, and the inability to pass any feces when at the privy. The two doses of salts, taken with a view to obviate this costiveness, in place of acting as a purgative, only aggravated the distress, by causing over excitation of the stomach and intestines. Then follow the rhubarb and calomel, which continued to add to the over stimulation of the stomach, which, being thus excited to the highest, discharged its contents, together with the secretions of its glandular appendages, the liver, spleen, and pancreas. Hence the vomiting, soon after swallowing the powder, of a large quantity of a greenish and bitter fluid: in fact, this over stimulation performs the office of an emetic, and thereby operates a partial revulsion of the morbid congestion. This partial revulsion procures a mitigation of the symptoms, and an alleviation of the sufferings of the patient, which lasts four days; at the end of which, the morbid sympathies, which have never been completely appeased and removed by any rational treatment, are aroused to an alarming activity, which may cause a rapid death through their excess, owing to the congestion and the disorganization of many viscera.

On the fifth day, when the practitioner is called, he finds the patient suffering greatly, tossing about the bed, and cannot remain a moment in one position; says that he feels as if his belly would burst; cannot bear the slightest pressure on the abdomen, which is very tense: all which denotes that inflammation has invaded not only the mucous, sensitive, digestive surface of its intestinal canal, which causes tumefaction of the viscera, but it has also propagated itself to the serous membranes, particularly the peritoneum; a membrane rich in nervous expansions, and of exquisitely acute sensibility when suffering under active inflammation. Hence the tension, and the impossibility to bear pressure on the abdomen. The headach denotes the participation of the sensorium, sympathetically irritated by the transmission to it of the sufferings of the viscera. The insatiable thirst denotes the inflammation predominating in the *ileum;* for, as there are two forms under which gastro-enteritis presents itself, I shall designate them here, in order to fix the seat of this terrible malady with as much precision as I am master of. The first form is that of predominancy of the gastric phlegmasia, which is characterized by gastric pain, by the aversion for ingesta, and the rejection of and the difficulty of supporting them. The second form is with predominancy of enteritis, or (as I understand it) of inflammation of the smaller intestine (ileum); for this insatiable thirst, and the rapidity with which the appropriated liquids are absorbed, characterize the second form: therefore I would infer that enteritis is predominating. The tongue white and coated with mucus, the mouth parched and dry, and the acid eructations, denote the complication of gastritis. The difficult micturition, and urine high coloured, denote the sympathetic participation of the urinary organs in this rapid and alarming phlegmasia. The pulse full, tense, and frequent, denotes the sympathetic participation of the heart, which is prevented thereby impelling the blood with measured force, and has its action precipitated. The temperature below the natural standard denotes the concentration of animal heat to be all in the inflamed viscera; too much vitality for the moment in the internal, and too little in the external, tissues. Same cause for the cold sweats. The nausea and vomiting of green bitter fluid denote association of gastritis. The want of sleep and great prostration denote the participation of the brain in the inflammatory suffering of the viscera.

I have thus gone through the whole group of symptoms detailed by the practitioner, and have endeavoured to analyze them according to the views of modern physiology as it applies to pathology, in order to fix, with as much certainty as possible, the attention of our medical brethren upon the nature and seat of a malady so fatal as that denominated dry bellyach of hot climates.

The intestines have for too long a time been considered, when in a state of disease, only as conduits, more or less dirty, more or less filled with acrid irritating matters; the vapours of which, it was pretended, arose to disturb the brain. Under these views, the diseases of the intestines were reduced to two forms: first, the superabundance and the too great frequency of the dejections; secondly, the retention, the rarity, the thinness of these same dejections: in short, the two prevailing morbid states were *diarrhœa* and *constipation.* When blood was joined to the stercoral matters voided in diarrhœa, the name of *dysentery* was given to it; and, when vomiting accompanied the dejections, it was called *cholera*, and all the different shades of *lienteria, cœliac flux, hepatic flux*, *melœna*, &c. The pains which appeared to have their seat in the intestines were called *colic, ileus*, or *iliac passion*, when the fecal matters were voided by the mouth. The name of *tenesmus* was given to the sensation of tension referred to the anus. The presence of *worms* was added to this nomenclature; as was also the *intestinal tympanitis, hemorrhoids,* &c.; and finally, came *enteritis.*

From a more correct acquaintance with this latter disease, we know that, with the exception of mechanical derangements, all the affections above enumerated, and even more, belong, directly or indirectly, to morbid irritation, to the inflammation of these viscera; that ulceration, schirrus, intestinal cancer itself, is a consequence of enteritis or colitis. It is thus that we see daily the important part which the intestines play in the *production of fevers.* In short, the knowledge of this important fact is one of the keys to physiological pathology, that "so soon as *local* morbid irritation arises to a certain degree, it repeats itself, and is propagated into other systems, or into apparatuses more or less distant from the primitive focus, and always without changing its nature."

This fact discovers the secret tie which links the slightest with the most serious maladies. It fills up an immense blank which existed in medical science from the remotest antiquity; it destroys that insulation of the divers shades of irritation, which may be regarded as the source of medical ontology; it reduces to their just value all the distinctions established by nosologists: take, for instance, the word dysenteria, composed of *dus* and *enteron*, what does this convey? *difficile intestinum*. Now, I would ask, in the name of common sense, how this difficulty has arisen in the intestine? what is its nature? and, if it be inflammation, why we are not told so.

It is with a view to the detection of like absurdities that these reflections are lengthened out, in order to recommend to our professional brethren to observe these maladies narrowly, by following an *external and visible* inflammatory irritation, abandoned to itself, from its commencement up to its highest degree of development; by observing it afterwards under the influence of modifiers, opposite in their effects; by comparing it in different sexes, and in climates the most opposite, we shall have the proof of what is meant to be expressed by the citation of the fact, "that, so soon as local morbid irritation arises to a certain degree of intensity, it propagates itself into other systems, or apparatuses more or less distant, and always without changing its nature." But as it is not so easy to make the same verification with respect to the morbid actions of organs deeply situated, we have only to exercise our memories in the practical observation of what passes on the outside of the body; and we shall find ourselves soon in a state to apply these observations to the viscera, the most deeply seated and the most concealed from our view, to the slightest shades of their irritation. I can assure our brethren, from experience, that they will find great pleasure in this species of study; for each succeeding day will dispel some doubt, will clear up some difficulty, and operate unexpected reconciliations on points that seemed before to be at variance. It is thus that we shall arrive at conviction; for it is impossible all at once, even for minds of deep penetration, to perceive at a glance all the consequences of a principle which is in itself no other than a conclusion resulting from the bringing together of an immense multitude of facts.

I hope, therefore, that the enlightened heads of the medical department will please to set our brethren employed in military-hospital practice to the observation of these principles in those terrible maladies called spasmodic cholera of India, dysenteria, colica pictonum, &c.; for, in my opinion, they are only shades of different degrees of intensity of gastro-intestinal inflammation, complicated at one time with peritoneal, at another time with hepatic, and at others with splenic, nephritic, cystic, and encephalic irritations.

In this case, so successfully treated by my friend, Mr. Barcome, I would, in place of colica pictonum, designate it

Gastro-entero-Peritonitis; and I will venture to affirm, that, had the case terminated fatally, dissection would have shown the ravages of the inflammation to have been confined chiefly to the viscera from which I have designated it, and that the colon was but little or nothing inflamed; for it is not until inflammation passes the ileo-cœcal valve that colitis attended with diarrhœa supervenes; whereas, in the case before us, costiveness prevailed, which is the characteristic of enteritis, or inflammation of the small intestines.

But, perhaps, one of the most important facts for medical science, to be gained from the contemplation of this case, is this, viz. that, in intestinal inflammation, local depletion, antiphlogistics, and emollients, are the real purgatives. For it was not until a reduction of the morbid congestion, effected by their means, was brought about, that any fecal matters could pass into the cœcum through the inflamed opening of the ileum; the drastics, given with a view to purge, only aggravated the inflammation, and added to the danger.

(Signed)

CHARLES DOYLE, M.D. P.M.O. Bermuda.

*Morbid Enlargement of the Scrotum.* By E. BARCOME, Esq.

William Kendell, native black of this colony, twenty-eight years old, is a well made, muscular man; occupation, that of a tailor; had always been healthy till within the last four years; was much exposed for several weeks, during martial law, as a private in the militia, at which time the right leg became painful, and swelled as high as the knee; soon after, the scrotum became similarly affected, and has increased to the present size. During this period has been subject to occasional attacks of intermittent fever.

Tumor broad at the bottom, and suspended by a narrow neck from the pubis; exterior covered with rugæ of different dimensions. Extremity of the prepuce has the appearance of a navel, from which the urine trickles. Does not evince any pain or suffering of any kind on exercising pressure; only inconvenience from its weight and bulk, which prevents his walking or leaving his house.

*Operation.*—Avoiding the corpora cavernosa, two oblique incisions were made, commencing at the opening of the prepuce, and continued along the sides of the tumor, meeting below the testes. The dissection was continued to the tunica vaginalis; on cutting into which, a large quantity of limpid fluid (twenty-five ounces) escaped from the left side.

The left testis was found to be schirrous, and was removed in the usual manner. The spermatic vein and artery were the only vessels necessary to be secured during the operation, at which but little blood was lost. The integuments spared by the scalpel were drawn over the parts exposed, and held together by means of stitches and adhesive plaster, assisted by a bandage.

The tumor, on examination after its removal, was found little vascular, and appeared to be composed of a bacon-like substance, intermixed with hydatids. Weight, twenty-five pounds.

July.—Twenty-six days after the operation, the parts were healed, and the patient is now able to walk about and attend to his business.

*Sudden Death, from Rupture of the Aorta Ascendens, &c.*
By E. Barcome, Esq.

September 26th, 1827.—Benjamin, a black man, a native of Bermuda, and sailor on board the brig Atlantic, from Newfoundland, is a short, muscular man; was blistered while at Newfoundland, and some pectoral medicines ordered for a cough, &c. On his arrival in this colony, which was ten or fifteen days previous to his death, he did not complain much, except of a slight pain and heat in the chest, with trifling cough and occasional constriction, of which no notice was taken, and he continued at his duty as a seaman on board. This morning, on attempting to assist in hoisting out a cask, he suddenly fell down, and expired.

*Dissection.*—On opening the thoracic cavity, a quantity of serous fluid, amounting to a pint, made its escape, and the pericardium was seen distended greatly, to about four or five times its natural size; on cutting it open, a quantity of dark grumous coagulated blood was observed therein, say three pounds. On removing the heart, with a considerable portion of its appendages, the aorta ascendens was found ossified and ulcerated, with a small rupture, about half an inch, where it emerges from the right ventricle, with appearances of high inflammation and aneurismal dilatation. The viscus itself rather smaller than natural, with its parietes much thickened, and the capacity of the ventricles, particularly of the right, greatly lessened; nothing remarkable in the valves. The pericardium had some few red spots on its internal tunic. The abdominal, with the pelvic, viscera, sound.

